# ASO Author Reflections: A Population-Based Study on Lymph Node Retrieval in Patients with Esophageal Cancer

**DOI:** 10.1245/s10434-018-6843-5

**Published:** 2018-10-08

**Authors:** L. R. van der Werf, B. P. L. Wijnhoven

**Affiliations:** Department of Surgery, Erasmus University Medical Centre, Rotterdam, The Netherlands

## Past

Oesophagectomy is the cornerstone of treatment for esophageal cancer. Extended lymphadenectomy may reduce locoregional failure and improve long-term survival. However, the value of lymph node dissection for locoregional tumor control has been debated since the introduction of neoadjuvant chemoradiotherapy. Neoadjuvant chemoradiotherapy leads to tumor shrinkage and results in a higher proportion of resections with tumor-negative resection margins and tumor-free lymph nodes.^1^

Studies have shown that the number of removed lymph nodes is associated with improved survival for patients who underwent surgery without neoadjuvant chemoradiotherapy. However, this positive correlation is lost in patients who underwent neoadjuvant chemoradiotherapy followed by surgery.^2^^,^^3^

Besides its role in locoregional tumor control, an extensive lymphadenectomy may improve tumor staging. Accurate pathological staging is essential for prognostication.^4^

## Present

In 2013, the number of lymph nodes removed was introduced as a quality indicator in the Dutch Upper gastrointestinal Cancer Audit (DUCA). This indicator is defined as “the percentage of patients with at least 15 retrieved LNs.” The purpose of our study was to evaluate trends in the number of retrieved lymph nodes and the proportion of patients with ≥ 15 LNs in the resection specimen.

Between 2011 and 2016, the median number of lymph nodes removed increased from 15 to 20. The percentage of patients with at least 15 lymph nodes removed also increased from 50 to 80% on a national level.^5^ There was a wide variation in the percentage of patients with at least 15 lymph nodes removed between hospitals (0–77%). In 2016, hospital variation decreased but was still considerable (between 50 and 98%).

## Future

Given the importance of lymph node retrieval for locoregional control and accurate staging, a higher number of lymph nodes removed will likely improve the quality of care. However, it remains unknown if the increased number of removed lymph nodes was due to extended surgery or more accurate pathological examination of the resection specimen. The DUCA does not contain long-term follow-up data. Hence, the study could not report on the association of number of lymph nodes removed and survival after neoadjuvant chemoradiotherapy.

An argument for the use of this indicator in clinical auditing is that there is a clinically relevant variation in outcomes between hospitals. This may lead to improvement initiatives in the hospitals underperforming. Continues use of this indicator in the DUCA may reduce variation and improve outcomes on a national level.
